# Quantitative Analysis of Global Proteome and Lysine Acetylome Reveal the Differential Impacts of VPA and SAHA on HL60 Cells

**DOI:** 10.1038/srep19926

**Published:** 2016-01-29

**Authors:** Xiaoyu Zhu, Xin Liu, Zhongyi Cheng, Jun Zhu, Lei Xu, Fengsong Wang, Wulin Qi, Jiawei Yan, Ning Liu, Zimin Sun, Huilan Liu, Xiaojun Peng, Yingchan Hao, Nan Zheng, Quan Wu

**Affiliations:** 1 Department of Hematology, Anhui Provincial Hospital, Anhui Medical University, Hefei, 230001, China; 2 Translational Medcine Advanced Institute, Tongji University, No.1239, Siping Road, Shanghai, 200092, China; 3 Jingjie PTM Biolab (Hangzhou) Co. Ltd, Hangzhou 310018, China; 4 Department of orthopaedics, Anhui Provincial Hospital, Anhui Medical University, Hefei, 230001, China; 5 School of Life science, Anhui Medical University, Hefei, 230032, China; 6 Central Laboratory of Medical Research Centre, Anhui Provincial Hospital, Anhui Medical University, Hefei, 230001, China

## Abstract

Valproic acid (VPA) and suberoylanilide hydroxamic acid (SAHA) are both HDAC inhibitors (HDACi). Previous studies indicated that both inhibitors show therapeutic effects on acute myeloid leukaemia (AML), while the differential impacts of the two different HDACi on AML treatment still remains elusive. In this study, using 3-plex SILAC based quantitative proteomics technique, anti-acetyllysine antibody based affinity enrichment, high resolution LC-MS/MS and intensive bioinformatic analysis, the quantitative proteome and acetylome in SAHA and VPA treated AML HL60 cells were extensively studied. In total, 5,775 proteins and 1,124 lysine acetylation sites were successfully obtained in response to VAP and SAHA treatment. It is found that VPA and SAHA treatment differently induced proteome and acetylome profiling in AML HL60 cells. This study revealed the differential impacts of VPA and SAHA on proteome/acetylome in AML cells, deepening our understanding of HDAC inhibitor mediated AML therapeutics.

Acute myeloid leukemia (AML) is a cancer of the myeloid line of blood cells, characterized by the rapid growth of abnormal white blood cells that accumulate in the bone marrow and interfere with the production of normal blood cells^1^. AML is the most common acute leukemia affecting adults with few effective treatment^2^. At present, chemotherapy is commonly used for AML treatment with the application of cytarabine and anthracycline^3^. However, because of the toxic effects of the therapy, chemotherapy could not be offered to the very elderly^4^. What’s worse, the cure result is not optimistic as complete cure of AML with chemotherapy is low, especially for the elderly^5^. Therefore, deeper study of the mechanism of AML genesis and development to find novel and more effective therapies is profound for the cure of AML.

Histone deacetylases (HDACs) are group enzymes which regulate chromatin remodeling and further impact gene expression through deacetylating histones in eukaryote^6^. Previous studies have found inhibitors of HDACs can cause growth arrest, differentiation and/or apoptosis of many tumors cells^7^,^8^. HDAC inhibitors are proving to be an exciting therapeutic approach to cancer and some HDAC inhibitors, such as suberoylanilide hydroxamic acid (SAHA) and valproic acid (VPA), have entered into clinical trials^6^,^9^,^10^. SAHA belongs to the hydroxamic acids group in the classes of compounds that are identified as HDAC inhibitors^11^. Its anti-tumor/cancer toxicity in lung cancer^12^,^13^, breast cancer^14^ and ovarian cancer^15^ have been reported in recent studies. SAHA even had been approved by the Food and Drug Administration (FDA) as HDACi drug for the treatment of refractory cutaneous T-cell lymphomas (CTCL)^16^. While VPA is a kind of short-chain fatty acids which acts as an HDAC inhibitor at relatively high concentrations^11^.

SAHA and VPA were both proved to be potential therapies for leucocythemia diseases. SAHA was verified to have activity against leukemia and other hematologic malignancies *in vitro* experiments^17^,^18^. Moreover, the clinic trial of SAHA in patients with advanced leukemias and myelodysplastic syndromes has also been reported and some optimistic results had been observed^18^,^19^. While VPA could induce the differentiation of carcinoma cells, transformed hematopoietic progenitor cells and leukemic blasts from myeloid leukemia patients, therefore it could be served as an effective drug for the treatment of different cancers including leucocythemia^19^. The clinic trial of the therapeutic effect of VPA in combination with other small molecules such as decitabine and 5-azacytidine in leucocythemia treatment had been conducted^20^,^21^,^22^. Though the anti-leucocythemia toxicity of SAHA and VPA had been confirmed, the underling mechanisms still need further investigation.

In this work, the global proteome and lysine acetylome of AML HL60 cell lines in response to SAHA and VPA treatment were intensively studied by the combination of SILAC labeling, high-efficiency acetylation enrichment and high-resolution LC-MS/MS analysis (Fig. 1). As a result, 5,775 proteins and 1,124 lysine acetylation sites were successfully identified. With advanced bioinformatics analysis, we aimed to explore the mechanisms underlying the SAHA or VPA inhibited AML development, which may promote the clinical trial and even application of SAHA and VPA in the therapy of AML as well as other subtypes of leucocythemias.

## Results

### Impacts of VPA and SAHA treatment on global proteome level in AML HL60 cells

**Figure 1:**
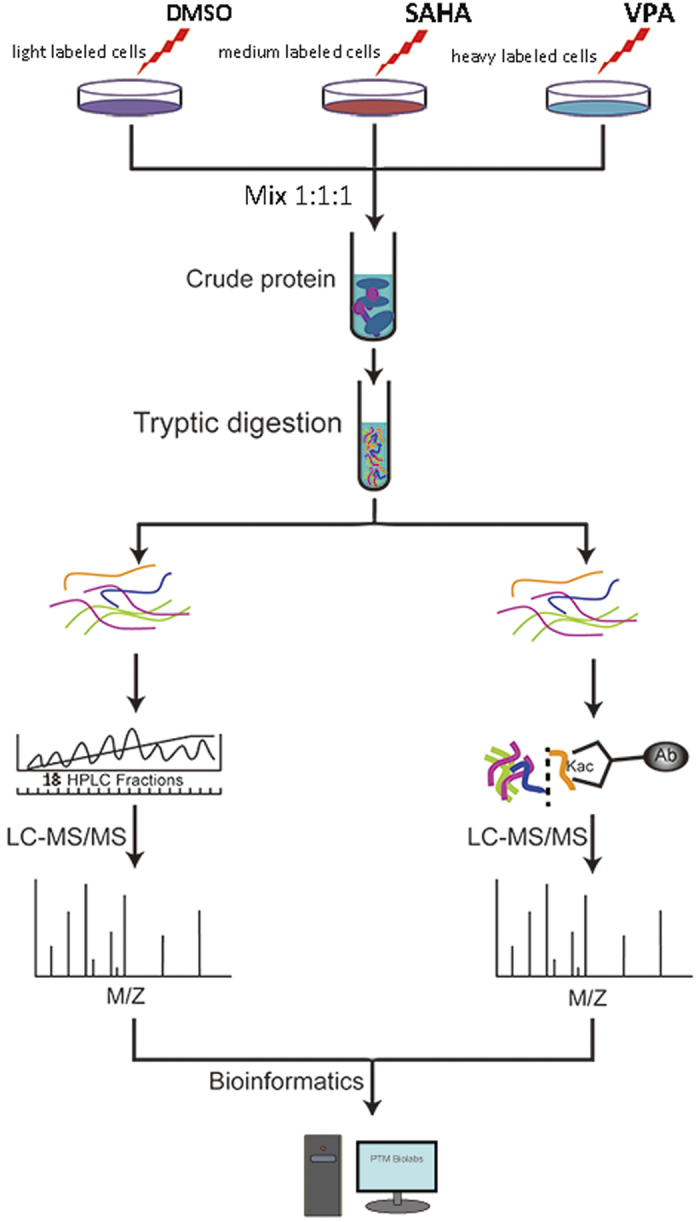
The workflow for the quantitative analysis of global proteome and lysine acetylome in VPA and SAHA treated HL60 cell lines. VP, valproic acid; SAHA, suberoylanilide hydroxamic acid.

By using the robust workflow by the integration of SILAC, basic HPLC fractionation and LC-MS/MS analysis (Fig. 1), 5,775 proteins from HL-60 were identified in response to VAP and SAHA treatment, among which 3,227 proteins were quantified. All the annotation and quantification information were presented in the Supplementary Table S1. With the threshold change fold >1.5, VPA treatment induced 785 differentially expressed proteins (359 up-regulated and 426 down-regulated) while SAHA treatment induced 775 differentially expressed proteins (323 up-regulated and 452 down-regulated).

To characterize the function and subcellular location distribution of these altered proteins, Gene Ontology (GO) function classification analysis and subcellular prediction were performed (Supplementary Figure S1). The GO-based classification on the ontology of biological process, cellular component and molecular function shown similar distribution between VPA and SAHA.

**Figure 2:**
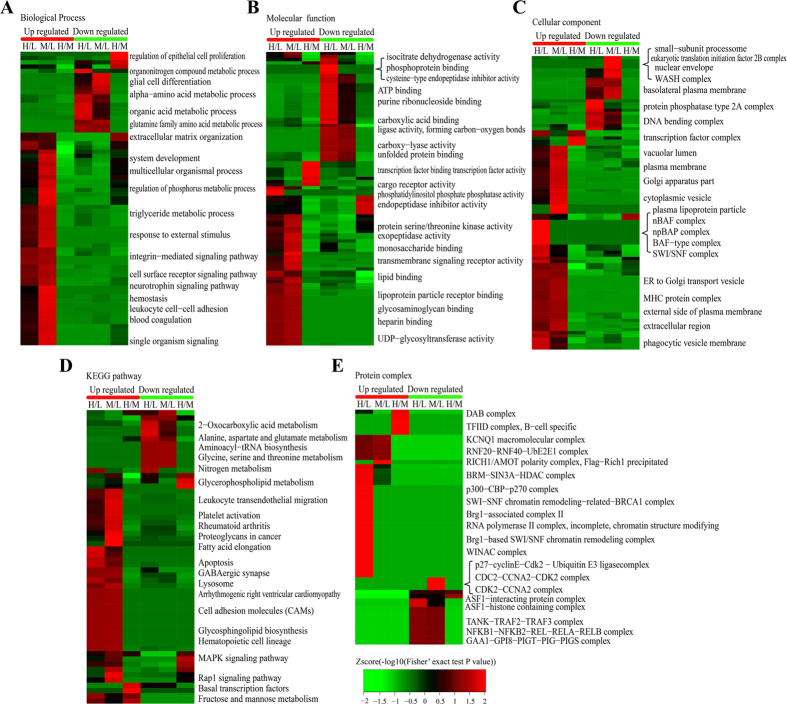
Enrichment and clustering analysis of the quantitative proteomics data sets in VPA and SAHA treated AML HL60 cells based on biological process (**A**), molecular function (**B**), cellular compartment (**C**), KEGG pathways (**D**), and protein complex (**E**). KEGG, Kyoto Encyclopedia of Genes and Genomes.

To reveal the nature of the differentially expressed proteins upon VPA and SAHA treatment, the GO enrichment based clustering analysis were carried out. The biological process was firstly investigated (Fig. 2A), it is found that the up-regulated proteins in response to VPA and SAHA treatment shown very similar enrichment results such as hemostasis, leukocyte cell-cell adhesion and blood coagulation, showing that VPA and SAHA were with similar impacts on the proteome of HL60 cells. In the down-regulated proteins, the enrichment results were also very similar between VPA and SAHA treatment except a few processes such as organonitrogen compound metabolic process which were induced only by VPA treatment. Molecular function-based clustering results were shown Fig. 2B. It is found that the phosphatidylinositol phosphate phosphatase activity and cargo receptor activity related proteins in up-regulated proteins and the cysteine−type endopeptidase inhibitor activity, phosphoprotein binding and isocitrate dehydrogenase activity in down-regulated proteins were enriched in VPA treatment. All of the other enriched molecular functions were similar in VPA and SAHA treatment. In the cellular component category a little more difference were found between VPA and SAHA treatment (Fig. 2C). In VPA treatment, plasma lipoprotein particle, and transcription regulation related protein complexes including npBAP complex, nBAF complex, BAF type complex and SWI/SNF complex were enriched in the up-regulated proteins, while SAHA treatment induced many membrane and vesicular related cellular components which involved in multi organelles. Vesicular transportation and transmembrane transportation probably be the high frequency events in HL60 cells incubated with SAHA. In the down-regulated proteins, the difference between VPA and SAHA treatment lies on nuclear envelope, small-subunit processome, eukaryotic translation initiation factor 2B complex, WASH complex, protein phosphatase type 2A complex and so on. However, the majority of the enrichment results were similar between treatment of the two HDACi.

To identify cellular pathways and the protein complex related with VPA and SAHA treatment, the clustering analysis based on KEGG pathway, protein complex and protein domain were performed (Fig. 2D,E). The results showed that the pathways of leukocyte transendothelial migration, lysosome, platelet activation and apoptosis were the dominant pathways enriched in the increased proteins in both VPA and SAHA treated cells while many amino acid metabolism related pathways including alanine, aspartate and glutamate metabolism, aminoacyl−tRNA biosynthesis, glycine, serine and threonine metabolism and nitrogen metabolism were notably enriched in both VPA and SAHA treated cells, hinting VPA and SAHA share common pathways in inhibiting HL60 cell proliferation.

In living organisms, the proteins assemble into various complexes to function better is a common phenomenon. Thus we analyzed the complex features of those enriched proteins induced by VPA and SAHA treatment (Fig. 2D). As the result shown, many chromosome templating and gene transcription regulated complex such as BRM−SIN3A−HDAC complex, p300−CBP−p270 complex and Brg1−based SWI/SNF chromatin remodeling complex were markedly enriched in the VPA induced up-regulated proteins. These complexes were extensively associated with histone lysine acetylation reaction, verifying the HDACs inhibitor role of VPA. RICH1/AMOT polarity complex (Flag−Rich1 precipitated) was the top significantly enriched complex in the SAHA induced up-regulated proteins. For the response proteins with decreased abundance, ASF1−interacting protein complex and ASF1−histone containing complex were significantly enriched upon VPA treatment while three cell cycle/cell division related complexes, namely p27−cyclinE−Cdk2 − Ubiquitin E3 ligase complex, CDC2−CCNA2−CDK2 complex and CDK2−CCNA2 complexes were markedly enriched upon SAHA treated cells. We naturally speculate that suppressed cell division was connected with the anti-leukaemia effects of SAHA.

In conclusion, GO enrichment and KEGG pathway based cluster analysis results indicated VPA and SAHA response proteins had similar impacts in AML HL60 cells, while the protein complex based enrichment shown lots of difference in the two HDACi treatment.

### Impacts of VPA and SAHA treatment on acetylome level in AML HL60 cells

Overall, 1,124 lysine acetylation sites in 720 proteins were identified in HL60 cell line, among which 1,089 acetylation sites in 686 proteins were quantitated. Then we use the quantification results of global proteome to normalize the acetylome quantification data. The final normalized quantification information of all the acetylation sites were shown in the Supplementary Table S2. With the threshold change fold >1.5, VAP treatment induced 186 up-regulated lysine acetylation sites on 164 proteins and 135 down-regulated lysine acetylation sites on 104 proteins while SAHA treatment induced 139 up-regulated sites on 124 proteins and 94 down-regulated sites on 88 proteins.

**Figure 3:**
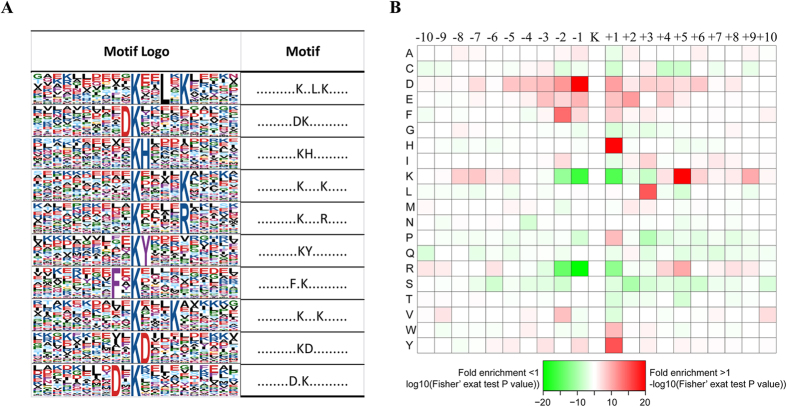
Properties of all the identified Kac peptides. (**A**) Acetylation motifs and conservation of acetylation sites. (**B**) Heat map of the amino acid compositions of the acetylated sites showing the frequency of the different types of amino acids around the acetylated lysine.

To characterize the possible specific sequence motifs surrounding acetylated lysine residues in HL 60 cells, we generated a type of sequence logo which computes the likelihood of amino acids being over- or under-represented at the positions surrounding the acetylation site (Fig. 3). Ten significantly enriched motifs were obtained from all the identified acetylated sites including Kac**L*K, DKac, KacH, Kac****K, Kac****R, KacY, F*Kac, Kac***, KacD, D*Kac (Kac represents the acetylated lysine and * represents a random amino acid residue, Fig. 3A). To determine if there are specific amino acids adjacent to acetylation lysines, we examined the amino acid sequences flanking acetylation sites by heat map (Fig. 3B). The two acidic amino acids aspartic acid (D) and glutamic acid (E) were overrepresented in multi positions surrounding Kac, suggesting their crucial role in sequence motifs surrounding Kac. Histidine acid (H) and tyrosine acid (Y) were overrepresented in the + 1 position of Kac. Besides, the two alkaline amino acid lysine (K) and arginine (R) seldom appeared in very near position such as ± 1, +2 position of Kac, but frequently appeared in the relative distant position such as −9, −8, +5 and + 9 position of Kac. Other amino acids whose frequencies of occurrence were relative high surrounding Kac were leucine (L,+3 position) and phenylalanine (F, −2 position).

**Figure 4:**
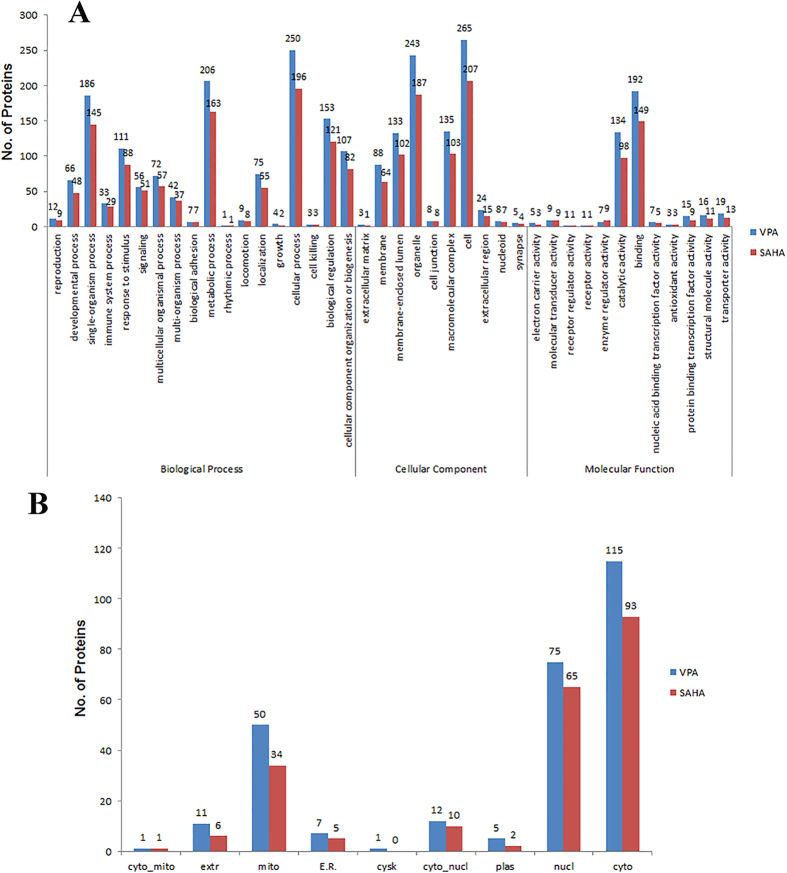
Gene ontology (GO) classification analysis (**A**) and subcellular location prediction (**B**) of the differentially expressed Kac proteins in VPA and SAHA treated AML HL60 cells.

**Figure 5:**
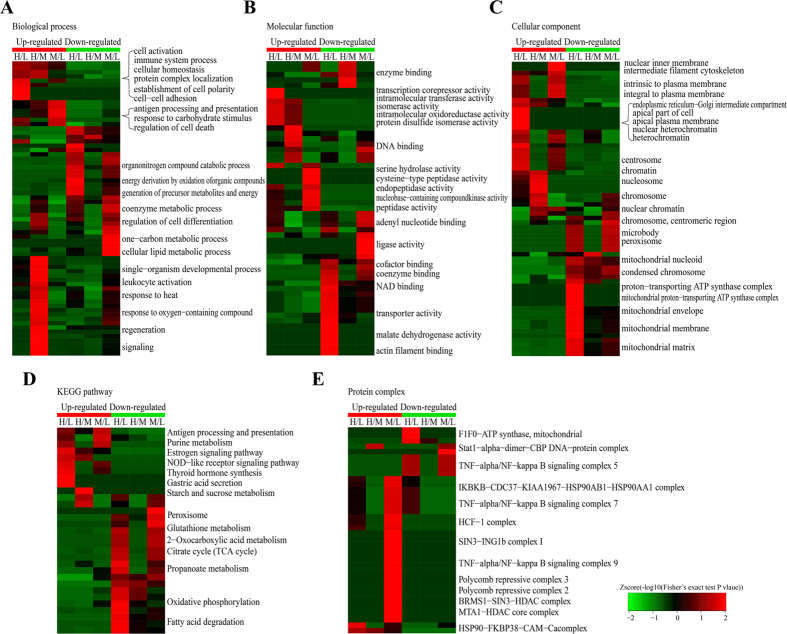
Enrichment and clustering analysis of the quantitative acetylome data sets in VPA and SAHA treated AML HL60 cells based on biological process (**A**), molecular function (**B**), cellular compartment (**C**), KEGG pathways (**D**), and protein complex (**E**).

To reveal the involved cellular processes and the subcellular location of the differentially expressed proteins in acetylation level upon SAHA and VPA treatment, the GO-based classification analysis and subcellular location prediction were conducted (Fig. 4). As shown in Fig. 4A, the distribution of biological process, cellular process and molecular function of the differentially expressed Kac proteins in VPA and SAHA induced cells were separately performed and compared. It is found that VPA and SAHA induced proteins shown very similar functionally distributions based on GO classification. The first three distributed biological processes are cellular process, metabolic process and single-organism process upon VPA and SAHA treatment. Cellular component classification result in cell, organelle, membrane-enclosed lumen, macromolecular complex and membrane related proteins as the dominant components. In the molecular function ontology, binding and catalytic activity related proteins were the overwhelming preponderant proteins for both VPA and SAHA as the percentage reached 46% and 31% (Fig. 5A). Subcellular location prediction result indicated that the differentially expressed Kac proteins were distributed in diverse subcellular location with cytoplasm (37%), nucleus (26%) and mitochondria (23%) as the prominent proteins for both VPA and SAHA treatment (Fig. 4B). In conclusion, the differentially expressed Kac proteins upon VPA and SAHA shown very similar distribution based on GO and subcellular location analysis.

As VPA is the inhibitor of HDAC1, 2 and 3 while SAHA is a much wider inhibitor which could inhibit HDAC1 to 10, the effect the two HDACi must be some different. To elucidate the cellular functions regulated by VPA and SAHA, the clustering analysis based on GO, KEGG pathway and protein complex were performed (Fig. 5). The GO-based clustering analysis includes biological process, cellular component and molecular function. As shown in Fig. 5A, in biological process clustering analysis, cell activation, immune system process, cellular homeostasis, protein complex localization processes, establishment of cell polarity, cell-cell adhesion were the top enriched processes in VPA induced Kac proteins with up-regulated level, while a number of antigen processing and presentation processes and processes of response to carbohydrate stimulus and regulation of cell death were enriched in SAHA induced Kac proteins with up-regulated level. In the down-regulated Kac proteins, Many carbon and energy metabolism associated processes were enriched toward VPA treatment while cofactor and coenzyme metabolic processes, regulation of cell differentiation, cell proliferation, lipid metabolic process and so on were markedly enriched in SAHA treatment.

On the ontology of molecular function (Fig. 5B), transcription corepressor activity, intramolecular transferase activity, isomerase activity, intramolecular oxidoreductase activity, protein disulfide isomerase activity and cysteine−type peptidase activity were prominent enriched in the VPA caused up-regulated Kac proteins. Upon SAHA treatment, serine hydrolase activity, nucleobase-containing compound kinase activity, endopeptidase activity and peptidase activity were markedly enriched, which was consistent with the result of cellular component enrichment and biological process enrichment analysis. As to proteins with decreased acetylation levels, significantly enriched terms were mostly concerned with cofactor binding, coenzyme binding, NAD binding, transporter activity, malate dehydrogenase activity and so on upon VPA treatment. However, with SAHA treatment, the down-regulated Kac proteins were intensively enriched to DNA binding, nucleotide binding and ligase activity related terms, which was in accordance with biological process and cellular component enrichment result. These suggested SAHA mainly interfere the acetylation events in nucleus.

In cellular component category (Fig. 5C), we noticed that endoplasmic reticulm-Golgi intermediate compartment, apical part of cell, apical plasma membrane, nuclear heterochromatin and heterochromatin were significantly enriched in the increased Kac proteins upon VPA treatment. Whereas upon SAHA treatment, the proteins with increased acetylation level were overrepresented to intermediate filament cytoskeleton, nuclear inner membrane, intrinsic to plasma membrane and integral to plasma membrane, implying membrane located proteins were hyper acetylated upon SAHA treatment. The proteins with decreased acetylation levels were markedly enriched to mitochondrion related components upon VPA treatment, which was an interesting phenomenon in AML HL60 cells. However, upon SAHA treatment, the proteins with decreased acetylated level were intensively enriched to nuclear, chromosome, peroxisome and microbody related components. These seemed conflict with the well-known HDAC inhibitor role of SAHA.

To investigate the differentially expressed Kac proteins involved pathways upon VPA and SAHA treatment, KEGG pathway enrichment based clustering analysis was conducted (Fig. 5D). Upon VPA treatment, the up-regulated Kac proteins were mainly enriched in Estrogen signaling pathway, thyroid hormone synthesis, gastric acid secretion and starch and sucrose metabolism. However, upon SAHA treatment, antigen processing and presentation, purine metabolism and NOD-like receptor signaling pathway were enriched. In the down-regulated Kac proteins, many carbon metabolism related pathways were markedly enriched to the VPA treatment such as TCA cycle, carbon metabolism, 2-oxocarboxyic acid metabolism, propanoate metabolism, pyruvate metabolism. Moreover, oxidative phosphorylation, fatty acid degradation were also enriched in VPA treatment. Whereas in SAHA treatment primary bile acid biosynthesis, peroxisome, glutathione metabolism were enriched as well as many carbon metabolism related pathways.

Protein complex assembling and disassembling could be regulated by acetylation and deacetylation. We performed the protein complex clustering analysis to found crucial protein complex influenced by VPA and SAHA treatment in AML-HL60 cells (Fig. 5E). SNW1 and HSP90-FKBP38-CAM-Ca complexes were the only two significantly enriched complexes in VPA treatment in the up-regulated Kac proteins. However, a number of markedly enriched complexes including TNF-alpha/NF-kappa B signaling complex 7, 8, 9 and 10, histone 3 complex, sin3 complex, msin3A complex, SIN3-HDAC-SAP30-ARID4 complex and BRMS1-SIN3-HDAC complex were obtained in SAHA treatment induced Kac proteins with decreased acetylation levels. The TNF-alpha, SIN3 and NCOR1 related complex were reported to have close relationship with leukemia. In the down-regulated Kac proteins, three complexes were significantly enriched in VPA treatment including XFIM complex, F1F0-ATP synthase, while five complexes were enriched in SAHA treatment including TNF-alpha/NF-kappa B signaling complex 5, RFC2-Rlalpha complex and dynein complex.

### Protein-protein interaction analysis reveals the impacts of VPA and SAHA treatment in AML HL60 cells

**Figure 6:**
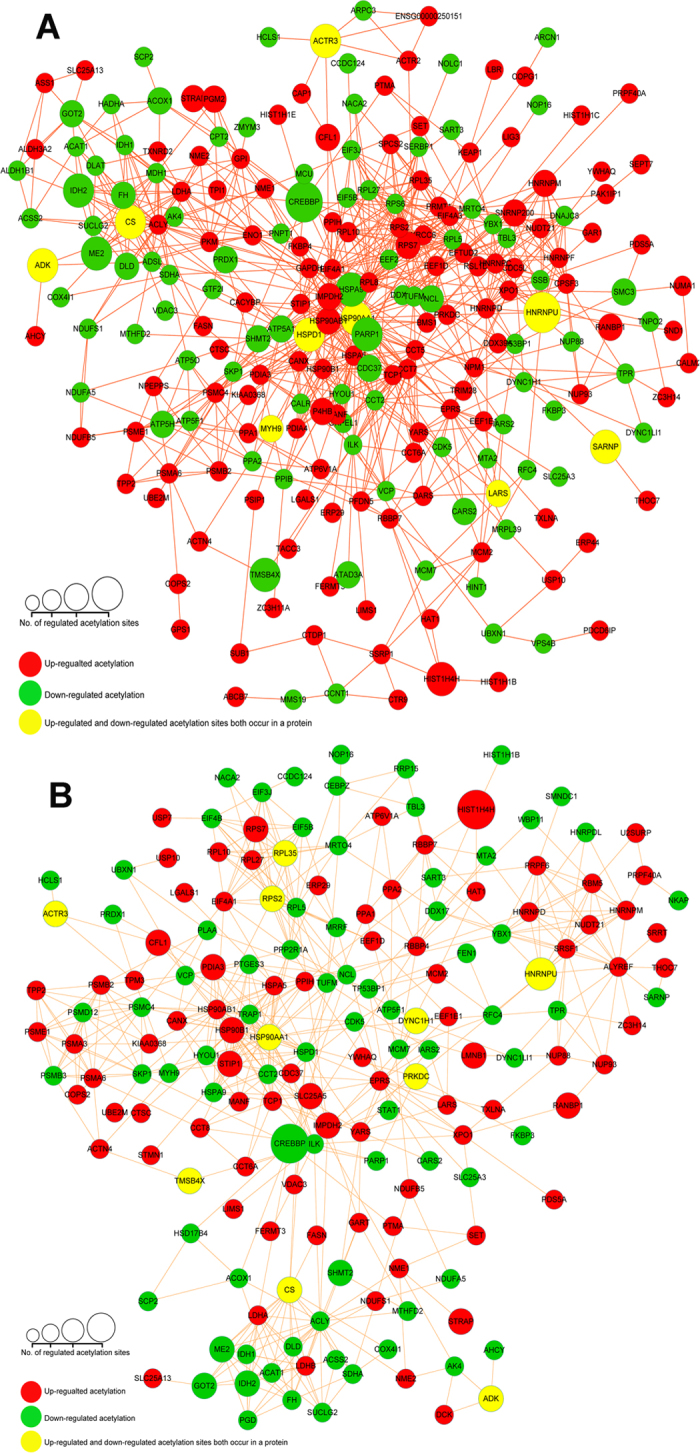
Protein-protein interaction network analyses for the different expressed 268 acetylated proteins upon VPA treated (**A**) and SAHA treated (**B**) in AML HL60 cells.

Previous study had shown lysine acetylation can affect protein-protein interaction by multiple mechanisms and interfere in subsequent biological processes^23^. Protein-protein interaction network analysis was conducted to disclose the important nodes and crucial interactions among the differentially expressed Kac proteins in VPA and SAHA treated AML-HL60 cells. The resulting interactome in VPA and SAHA induced Kac proteins were presented in Fig. 6A,B, respectively. There are 234 nodes and 814 interactions in the interactome of VPA induced Kac proteins, and 188 nodes and 542 interactions in the interactome of SAHA induced Kac proteins. The top ten nodes in VPA-induced interactome are HSP90AA1, HSP90AB1, ACLY, HSPA5, EFTUD2, RPL5, RPS6, CCT5, EEF2 and CCT7 while the top ten nodes in SAHA-induced interactome are HSP90AA1, ACLY, HSP90AB1, RPL5, HSPA5, HSPD1, RPS2, ALYREF, CS and TCP1, among which five were overlapped with VPA-induced interactome, showing the similarity of the two interactome. But the interaction targets or interaction pairs of these central nodes were not always the same. For example, we hypothesis VPA and SAHA treatment affected the acetylation level of these central nodes with possible similar mechanism in AML-HL60 cells. But the subsequent cascade acetylation events and downstream signaling pathway may be different, thus the differential proteome and acetylome were presented in the aforementioned enrichment analysis (Figs 2 and 5). Anyway, these crucial nodes played central roles in the network and acetylation events in AML-HL60 cells undoubtedly. Deeper studying the acetylation changes of these crucial nodes and their target proteins with biochemical and immunohistochemical techniques may promote to reveal the mechanism underlying VPA and SAHA anti-hematological malignant diseases effects, which is of potential clinical guiding significance.

## Discussion

### Deciphering the protective roles of VPA and SAHA against AML at proteome level

Previous studies have shown the inhibition effects in AML of VPA and SAHA as well as other HDACis. But the proteomic studies of the therapeutical role of VPA and SAHA in AML were still limited. In the present study, we found the response proteins of VPA and SAHA treatment in AML HL 60 cells showed a wide range of function and subcellular distribution. As both of VPA and SAHA were HDAC inhibitors, bioinformatics analysis of functional classification and enrichment-based clustering analysis showed that differentially expressed proteins with very similar distribution of biological process, functions and participated pathways were obtained in global proteome level (Supplementary Fig. S1 and Fig. 2). However, there are still quite a few differences identified between the two treatments, implying there may be some different mechanisms in the anti-AML effects of VPA and SAHA on proteomic level.

Upon VPA treatment, we observed many chromosome templating and gene transcription regulated complex were markedly enriched (Fig. 2E) to the up-regulated proteins, especially the SWI/SNF related complexes. SWI/SNF-like Brg1/Brm-associated factor (BAF) complex plays important roles in controlling cell proliferation and differentiation, it shows anti-proliferative effects through its chromatin-remodeling activity by inducing nucleosome conformation and may function as a tumor suppressor^24^,^25^,^26^. Many other identified SWI/SNF complex related components were also reported participated in tumor and cancer inhibition and loss of SWI/SNF function has been associated with malignant transformation^26^,^27^. VPA may activate the expression of SWI/SNF complex related proteins, and then show cell growth and proliferation inhibition effects in AML HL60 cells. The enriched BAF complex and SWI/SNF complex in cellular component analysis (Fig. 2C,E) as well as enriched apoptosis pathway in KEGG pathway analysis (Fig. 2D) among the up-regulated proteins consolidated this speculation. Moreover, the term cysteine−type endopeptidase inhibitor activity involved in apoptotic process was markedly enriched to the down-regulated proteins upon VPA treatment (Fig. 2B). Cysteine−type endopeptidase is an important endopeptidase mediating cell apoptosis^28^, VPA activated other apoptosis pathways in leukemia cells had been reported^29^,^30^. Dispelling the inhibitor of cysteine−type endopeptidase and activated cell apoptosis may be another apoptosis approach in AML, which may be used as therapy target of AML.

Compared with VPA treatment, much more diverse cellular processes and metabolism pathways were activated by SAHA treatment. Immune/disease response, signal and development related processes and pathways were the top three conspicuous enriched groups in the up-regulated proteins upon SAHA treatment (Fig. 2A,D). HDACs were involved in both innate immunity and adaptive immunity extensively^10^,^31^,^32^. SAHA treatment may inhibit HDACs and promote the expression of immune and disease response related proteins, and then activated immune system participated in anti-tumor/cancer processes in HL60 cells. Moreover, much vesicular transportation related organelles or components were also conspicuously enriched in the up-regulated proteins (Fig. 2B), suggesting SAHA treatment accelerated vesicular transportation in HL60 cells. The relationship between SAHA and vesicular transportation was rarely studied and further experiments were needed to uncover the biological significance of the accelerated vesicular transportation in SAHA treated HL60 cells.

In the down regulated proteins, three cell cycle and cell division related complex were significantly enriched (Fig. 2E). CCNA2 binds and activates CDC2 or CDK2 kinases and thus promotes both cell cycle G1/S and G2/M transitions^33^. Previous study found SAHA can inhibit cancer progression through arresting cells in G_1_/S or G_2_/M in ovarian cancer cell line^34^, chondrosarcoma cell lines^35^ and pancreatic cancer cells^36^. The arresting of cell G_1_/S or G_2_/M transition may be an extensive approach in SAHA inhibited cancer cell proliferation. Furthermore, amino acid metabolism related pathways (Fig. 2E) were also markedly enriched in the SAHA treatment induced down-regulated proteins, which is similar with VPA treatment. There may be some common approaches in the VPA and SAHA inhibited AML cell proliferation.

### Parsing the anti-AML role of VPA and SAHA at acetylome Level

The anti-tumor/cancer effects of various HDACs were closely connected with their protein acetylation regulation role^10^. We performed the quantitative acetylome study of VPA and SAHA treated HL60 cells to study the mechanism of VPA and SAHA inhibited HL60 cell proliferation at acetylome levels. Upon VPA treatment, the cytoskeleton and microtubule related processes were also significantly enriched in the increased Kac proteins (Fig. 5C). The increased protein acetylation may affect cytoskeleton dynamic assembling and disassembling equilibrium. Previous study had reported VPA treatment disrupted microtubules and microfilaments and further affected cell morphology and motility^37^. Here we hypothesized a possible mechanism of VPA disturbed cytoskeleton dynamic regulation. Dysfunctional cytoskeleton may involve in AML cell proliferation suppression.

In the clustering analysis of down-regulated Kac proteins upon VPA treatment, we observed that many carbon metabolism and energy production related processes and pathways were markedly enriched (Fig. 5A,D). In addition, cellular component analysis indicated that numerous mitochondria located proteins showed decreased acetylation level (Fig. 5C). Obviously, VPA treatment decreased the acetylation levels of carbon metabolism and energy production related proteins in HL60 cells, which seemed conflict with the deacetylase inhibitor role of VPA. However, VPA generally work on class I and class IIa deacetylases^32^, the regulation of the acetylation level of energy metabolism related proteins was generally performed by class III deacetylases (Sirts), which mainly located in mitochondria^38^. Acetylation regulates metabolic enzymes by multiple mechanisms, including via enzymatic activation or inhibition and influencing protein stability^39^. VPA may decrease the acetylation level of these energy production related enzymes by HDACs mediated indirect approaches, and Sirts may also be involved. But the detailed signal cascade pathways need to be further studied. Metabolism enzymes with decreased acetylation level may mediate AML cell death through disturbing energy supply.

Upon SAHA treatment, many antigen processing/presentation and immune/disease response process and pathways were enriched to the quantity of increased acetylation level (Fig. 5A), indicating antigen presentation and immune response related proteins were hyper acetylated upon SAHA treatment. Previous studies reported that some HDACs might affect neoplastic growth and survival by regulating host immune responses through multi approaches including augment the immunogenicity of tumor cells to make them more attractive immune targets, altering immune cell activity and/or cytokine production^39^,^40^,^41^. We speculate hyper acetylated proteins in the cell surface make cancer cell be more attractive to various killer cells. But the other two immunity response approaches may also involve in SAHA mediated AML cell death process.

More importantly, in the up-regulated Kac proteins upon SAHA treatment, a number of protein complexes such as TNF-alpha/NF-kappa B signaling complex 7, 8, 9 and 10, histone 3 complex, sin3 complex, msin3A complex, SIN3-HDAC-SAP30-ARID4 complex and BRMS1-SIN3-HDAC complex (Fig. 5E). It is reported that TNF-alpha, SIN3A and SIN3-HDAC complex have close relationship with leukemia. Therefore, the results indicated that the mechanism of SAHA treatment to AML may lies on these complexes. VPA has weaker regulation on lysine acetylation compared with SAHA, but a number of similar complexes including BRM-SIN3A-HDAC complex, BRG1-SIN3A complex, BRM-SIN3A complex, BRG1-SIN3A-HDAC complex, which may regulated by VPA at proteome-scale.

Recently, Choudhary and colleagues reported a systematic study of 19 HDACi on lysine acetylation in HeLa cells, among the 19 HDACi, SAHA and VPA were also included^42^. Therefore, we compared our results with the published data to see if SAHA and VPA treatment leads to similar changes at acetylation levels in different cell lines (Supplementary Table S3). It was found that among the identified lysine acetylation sites in this work, 583 were also reported in the published data. For the quantification results, we found that quite a number of acetylation sites shown similar quantification ratio in the two studies. For example, Endoplasmin K265, Histone H1.4 K16, Histone H4 (K12, K8), P300 (K1558, K1560) were all up-regulated in the two studies for both SAHA and VPA. Meanwhile, some acetylation sites were found both down-regulated in the two studies, such as ATP synthase subunit alpha (K161, K498, K539) and ATP synthase subunit d (K63, K85) for both SAHA and VPA. However, there are still some acetylation sites shown different alteration trends. For example, Histone H3 (K27, K36), P300 (K1546, K1542), acyl-CoA-binding protein (K19, K55, K77) were quantified as up-regulated in this work but not changed or down-regulated in the published data for both SAHA and VPA. According to our previous study, these sites were also up-regulated upon SAHA treatment in non-small cell lung cancer A549 cell line^13^. As in published data, Hela cell was used, these differences may due to difference between cell lines.

## Methods

### Reagents and materials

Media and heavy lysine ([^2^H_4_]-L-lysine and [^13^C_6_, ^15^N_2_]-L-lysine) were purchased from Cambridge Isotope Laboratories (Andover, MA). The reagents of trichloroacetic acid (TCA), dithiothreitol (DTT), iodoacetamide (IAA), trifluoroacetic acid (TFA), ammonium bicarbonate (NH4HCO3) and ammonium formate were all purchased from Sigma-Aldrich (St. Louis, MO, USA). Acetonitrile (ACN) and pure water were obtained from Thermofisher (Waltham, MA, USA). 2-D Quant kit was obtained from GE Healthcare (Buckinghamshire, United Kingdom). Trypsin was from Promega (Fitchburg, WI, USA). Protease inhibitor cocktail set III was obtained from Millipore (Billerica, MA, USA). Anti-acetyllysine antibody agarose beads were purchased from PTM Biolabs (Cat. No. 104, PTM Biolabs, Hangzhou, China). Sep-Pak C18 SPE columns were purchased from Waters (Framingham, USA).

### Cell culture and SILAC labeling

AML HL60 cells were maintained in SILAC Protein Quantitation Kit (Invitrogen, Carlsbad, CA) according to manufacturer’s instructions. Briefly, cells were maintained in SILAC-1640 culture medium supplemented with 10% FBS (Life Technologies, Grand Island, NY) at 37 °C in humidified atmosphere with 5% CO_2_, with medium renewal at every 2 or 3 days depending on cell density. The cells were supplemented with unlabeled L-lysine (light labeling), “heavy (^2^H_4_)” form of L–lysine (medium labeling) or “heavy (^13^C_6_) and heavy (^15^N_2_)” form of L–lysine (heavy labeling) for stable isotope labeling, respectively. To achieve complete isotope incorporation, the HL-60 cells were cultured in medium for at least 8 generations as previously described^43^. The media and heavy labeling efficiency was evaluated by mass spectrometer analysis to a confirmed >99% labeling efficiency. Then, cells were continuously expanded to obtain large cell populations. Afterward, the medium labeled HL-60 cells were treated with 205 μM SAHA and the heavy labeled HL-60 cells were treated with 5 mM VPA for 8 h, while the light labeled HL-60 cells were treated with the same volume of DMSO for the same time. Finally, cells in each labeled pool were harvested and combined with equal amount.

### Sample preparation and HPLC fractionation

The cell protein extraction and digestion were carried out as previously described^44^. Briefly, cells were suspended in ice-cold lysis reagent (8 M urea, 5 mM DTT, 1% (v/v) protease inhibitor cocktail) for 10 min with occasional sonication. Cell lysates were centrifuged at 16,000 g at 4 °C for 20 min, and the resulting supernatants were collected. The protein concentration was determined with the 2-D Quant kit. Afterwards, the proteins were precipitated with 20% TCA overnight at 4 °C and resulting precipitate was desalted for 3 times with ice-cold acetone.

Dried protein pellets were re-suspended in 100 mM NH_4_HCO_3_ and digested with trypsin at an enzyme-to-substrate ratio of 1:50 for 12 h at 37 °C. Then the peptides were reduced with DTT and alkylated with IAA. To ensure complete digestion, the second digestion was conducted by adding trypsin at enzyme-to-substrate ration of 1:100 for 4 h at 37 °C as previously descripted^45^.

To lower the complexity of the peptides mixture and enhance the accuracy and throughout of protein identification, the protein digestion was fractionated by high pH reverse-phase HPLC using Agilent 300Extend C18 column (5 μm particles, 4.6 mm ID, 250 mm length) with solvent A of 98% H_2_O and 2% acetonitrile containing 10 mM ammonium formate (pH 10) and solvent B of 2% H_2_O and 98% acetonitrile containing 10 mM ammonium formate. The LC gradient was from 2% to 60% solvent B for 80 min to generate 80 fractions with 1 min per fraction and then combined into 18 fractions for the global proteome analysis as previously descripted^46^ and 8 fractions for lysine acetylome analysis. The fractionated sample was dried by vacuum centrifugation and stored at −20 °C for further usage.

### Affinity enrichment of lysine acetylated peptides

Acetylated peptides were enriched according to Chen *et al.*^47^ In brief, the tryptic peptides was re-dissolved in NETN buffer NETN buffer (100 mM NaCl, 1 mM EDTA, 50 mM Tris-HCl, 0.5% NP-40, pH 8.0) and incubated with anti-acetyllysine antibody agarose conjugated beads (PTM Biolabs, Hangzhou, China) in a ratio of 15 μL beads/mg protein at 4 °C overnight with gentle shaking. After incubation, the beads were washed four times with NETN buffer and twice with purified water. The bound peptides were eluted with 1% TFA and dried under a vacuum.

### LC-MS/MS analysis

Peptides were dissolved in solvent A (0.1% FA in 2% ACN, 98% H_2_O), directly loaded onto a reversed-phase pre-column (Acclaim PepMap100 C18 column, 3 μm, 75 μm × 2 mm, 100 Å, Thermo Scientific). Peptide separation was performed using a reversed-phase analytical column (Acclaim PepMap RSLC C18 column, 50 μm × 15 mm, 2 μm, 100 Å, Thermo Scientific). The gradient was comprised of an increase from 6% to 22% solvent B (0.1% FA in 98% ACN) for 24 min, 22% to 36% for 10 min and climbing to 80% in 3 min then holding at 80% for the last 3 min, at a constant flow rate of 300 nl/min on an EASY-nLC 1000 UPLC system, the resulting peptides were analyzed by Q Exactive^TM^ Plus hybrid quadrupole-Orbitrap mass spectrometer (Thermo Scientific).

The peptides were subjected to NSI source followed by tandem mass spectrometry (MS/MS) in Q Exactive^TM^ Plus coupled online to the UPLC. Intact peptides were detected in the Orbitrap at a resolution of 70,000. Peptides were selected for MS/MS using NCE setting as 28; ion fragments were detected in the Orbitrap at a resolution of 17,500. A data-dependent procedure that alternated between one MS scan followed by 20 MS/MS scans was applied for the top 20 precursor ions above a threshold ion count of 1.5E4 in the MS survey scan with 15.0 s dynamic exclusion. The electrospray voltage applied was 2.0 kV. Automatic gain control (AGC) was used to prevent overfilling of the ion trap; 5E4 ions were accumulated for generation of MS/MS spectra. For MS scans, the m/z scan range was 350 to 1600.

### Database processing

The protein and acetylation sites identification and quantification were performed through MaxQuant with an integrated Andromeda search engine (version 1.4.1.2). Tandem mass spectra were searched against SwissProt human database (20,274 sequences) concatenated with reverse decoy database and protein sequences of common contaminants. Trypsin/P was specified as cleavage enzyme allowing up to 3 missing cleavages, 4 modifications per peptide and 5 charges. Mass error was set to 5 ppm for precursor ions and 0.02 Da for fragment ions. Carbamidomethylation on Cys was specified as fixed modification and oxidation on Met, acetylation on lysine and acetylation on protein N-terminal were specified as variable modifications for lysine acetylation searching. False discovery rate (FDR) thresholds for protein, peptide and modification site were specified at 0.01. Minimum peptide length was set at 7. Acetylation sites identifications with localization probability less than 0.75 or from reverse and contaminant protein sequences were removed. Protein quantification was based on the average ratios of peptide pairs, which was observed in the full MS spectra. To minimize the systematic errors introduced by the Bradford assay and sample mixing, the normalization was done using a multiple-point normalization strategy according to previous report^48^.

### Protein classification and subcellular location prediction

Protein function classification was performed according to the Gene Ontology (GO)^49^ database annotation and Kyoto Encyclopedia of Genes and Genomes (KEGG)^50^ database annotation of the different expressed proteins. Wolfpsort (a subcellular localization predication software) was selected to perform the subcellular localization analysis^51^.

### Motif analysis

Soft motif-x^52^ was used to analysis the model of sequences constituted with amino acids in specific positions of acetyl-21-mers (10 amino acids upstream and downstream of the site) in all protein sequences. And all the database protein sequences were used as background database parameter, other parameters with default.

### Functional enrichment analysis

DAVID Bioinformatics Resources 6.7 version was selected to perform the Enrichment analysis^53^. A two-tailed Fisher’s exact test was employed to test the enrichment of the protein-containing IPI entries against all IPI proteins. Correction for multiple hypothesis testing was carried out using standard false discovery rate control methods. A corrected p-value <0.05 is considered significant for all the enrichment analysis^51^.

### Enrichment-based clustering analysis

All the substrates categories obtained after enrichment were collated along with their P values, and then filtered for those categories which were at least enriched in one of the clusters with P value <0.01. This filtered P value matrix was transformed by the function x = −log10 (P value). Finally these x values were z-transformed for each category. These z scores were then clustered by one-way hierarchical clustering (Euclidean distance, average linkage clustering) in Genesis. Cluster membership was visualized by a heat map using the “heatmap.2” function from the “gplots” R-package.

### Protein-protein interaction analysis

The search tool for the Retrieval of Interacting Genes/Proteins (STRING) database was used to annotate functional interactions of all the identified acetylated proteins by calculated their confidence score. Only high-confidence interactions (score >7) between the acetylated proteins and high confidence interactions (with score >0.7) in the STRING database were fetched for the analysis. Cytoscape software (version 3.0.1) was used to visualize the interaction network^54^.

## Additional Information

**How to cite this article**: Zhu, X. *et al.* Quantitative Analysis of Global Proteome and Lysine Acetylome Reveal the Differential Impacts of VPA and SAHA on HL60 Cells. *Sci. Rep.* **6**, 19926; doi: 10.1038/srep19926 (2016).

## Supplementary Material

Supplementary Information

Supplementary Table S1

Supplementary Table S2

Supplementary Table S3
