# Supplementary material for: Quantitative Analysis of Global Proteome and Lysine Acetylome Reveal the Differential Impacts of VPA and SAHA on HL60 Cells
Source: Sci Rep. 2016 Jan 29;6:19926. doi: 10.1038/srep19926 (PMC4731804; doi:10.1038/srep19926)

**Quantitative Analysis of Global Proteome and Lysine Acetylome Reveal the Differential Impacts of VPA and SAHA** **on HL60 Cells**

Xiaoyu Zhu^1,7^, Xin Liu^1,7^, Zhongyi Cheng^2^, Jun Zhu^3^, Lei Xu^4^, Fengsong Wang^5^, Wulin Qi^3^, Jiawei Yan^1^, Ning Liu^1^, Zimin Sun^1^, Huilan Liu^1^, Xiaojun Peng^3^, Yingchan Hao^6^, Nan Zheng^6^, Quan Wu^6^

^1^ Department of Hematology, Anhui Provincial Hospital, Anhui Medical University, Hefei, 230001, China

^2^ Translational Medcine Advanced Institute, Tongji University, No.1239, Siping Road, Shanghai, 200092, China

^3^ Jingjie PTM Biolab (Hangzhou) Co. Ltd, Hangzhou 310018, China

^4^ Department of [orthopaedics](javascript:void(0);), Anhui Provincial Hospital, Anhui Medical University, Hefei, 230001, China

^5^ School of Life science, Anhui Medical University, Hefei, 230032, China

^6^ Central Laboratory of Medical Research Centre, Anhui Provincial Hospital, Anhui Medical University, Hefei, 230001, China

^7^ Xiaoyu Zhu and Xin Liu contributed equally to this study and should be considered as co-first authors.

Correspondence and requests for materials should be addressed to

Q.W. (powerwoo01@ahmu.edu.cn, Ph: +86-551-62283574, fax: +86-551-62283292)

To characterize the function and subcellular location distribution of these altered proteins, Gene Ontology (GO) function classification analysis and subcellular prediction were performed. The GO-based classification was performed on the ontology of biological process, cellular component and molecular function. As shown in **Supplementary Figure S1A**, the classiﬁcation results for biological processes showed that the differentially expressed proteins participated in diverse biological processes upon VPA treatment. Among them, cellular process, metabolic process, single-organism process and biological regulation related proteins accounted for relative higher proportion compared with other process related proteins. On the ontology of cellular component, cell and organelle related proteins accounted for the largest portion, whose percentage was 33% and 28%, respectively. Moreover, the membrane (14%), macromolecular complex (11%) and membrane-enclosed lumen (10%) related proteins were also identified. Extracellular region and other proteins only accounted for very small part of among all the different expressed proteins. Molecular function analysis result indicated the majority proteins were binding and catalytic activity related proteins, whose percentage was 46% and 31%, respectively. Upon SAHA treatment, GO classification analysis of the differentially expressed proteins showed highly similar results as VPA, even the proportion of each function category was almost the same (**Supplementary Fig. S1C**), showing the similar effect of VPA and SAHA to HL60 cells in proteome level.

Subcellular location were also performed to compare the effect of SAHA and VPA. Again, the location shows very similar results upon VPA and SAHA treatment (**Supplementary Fig. S1B** and **D**). The majority differentially expressed proteins were localized in nucleus (33% for VPA and 32% for SAHA) and cytoplasm (32% for both VPA and SAHA), following by the mitochondria, plasma and extracellular located proteins for both VPA and SAHA treatment.

**Figure legend**

**Figure S1** Gene ontology (GO) classification analysis and subcellular location prediction of the different expressed proteins in VPA (A and B) and SAHA (C and D) treated AML HL60 cells. Cyto_nucl, cytosol nuclear; nucl, nulear; E.R., endoplasmic reticulum; cyto, cytosol; mito, mitochondria; extr, extracellular; plas, plasma membrane.

Figure S1


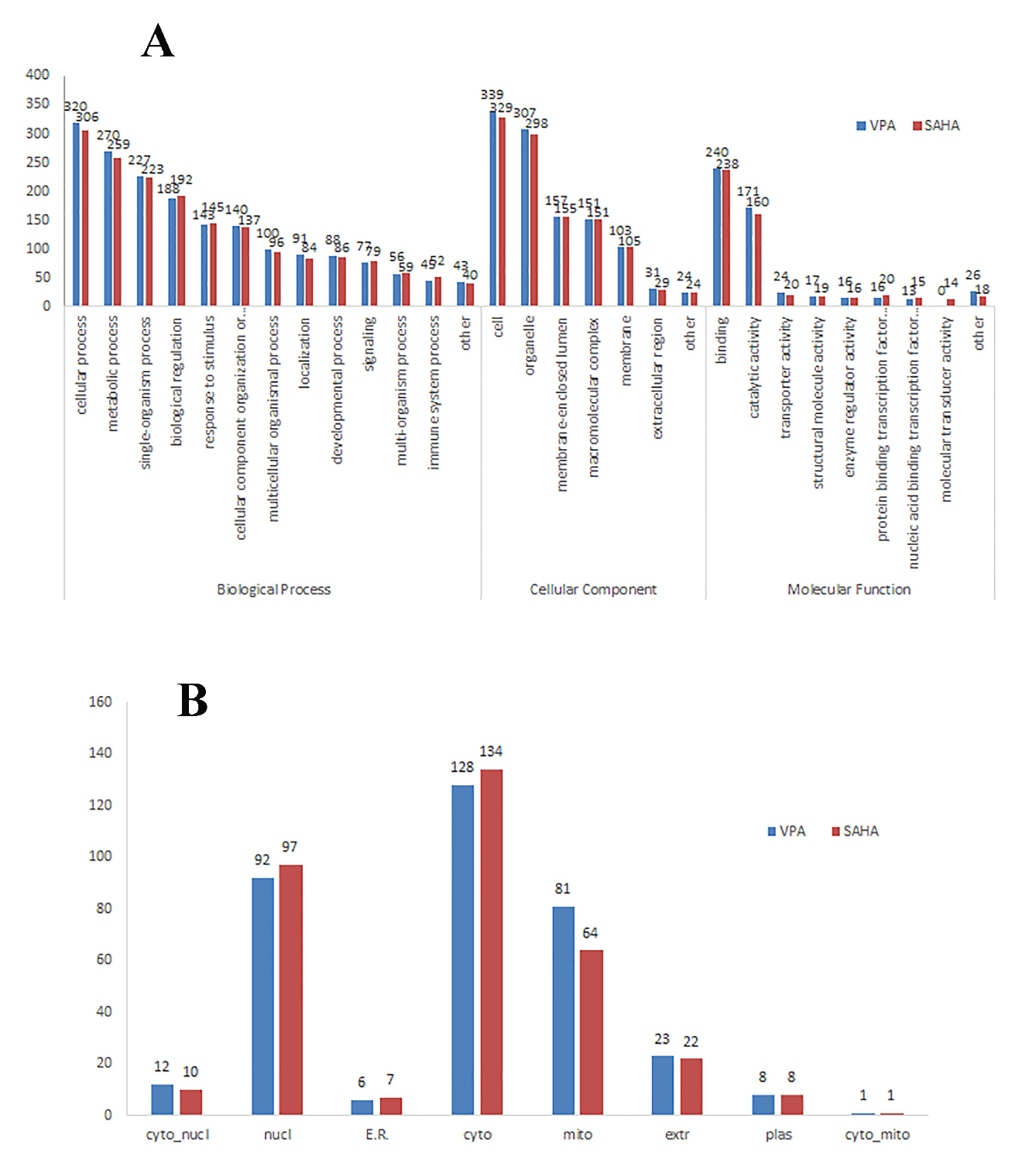

Supplement: Supplementary Information [file srep19926-s1.doc]
